# Diverse Effect of Vitamin C and N-Acetylcysteine on Aluminum-Induced Eryptosis

**DOI:** 10.1155/2021/6670656

**Published:** 2021-01-12

**Authors:** Ali Reza Zangeneh, Mohammad Ali Takhshid, Reza Ranjbaran, Mahsa Maleknia, Mohammad Hassan Meshkibaf

**Affiliations:** ^1^Department of Clinical Biochemistry, Fasa University of Medical Sciences, Fasa, Iran; ^2^Diagnostic Laboratory Sciences and Technology Research Center, Faculty of Paramedical Sciences, Shiraz University of Medical Sciences, Shiraz, Iran

## Abstract

**Purpose:**

The role of oxidative stress in Aluminum (Al)-induced apoptotic effects has been investigated and suicidal death of erythrocytes, eryptosis, is characterized by cell shrinkage and phosphatidylserine externalization (PSE) at the surface of the erythrocyte cell membrane. Eryptosis is stimulated by an increase in cytosolic Ca^2+^ concentration and reactive oxygen species (ROS). This ex vivo study was conducted to evaluate the effect of well-known antioxidants including vitamin C (vit C) and N-acetylcysteine (NAC), against Al-induced hemolysis and eryptosis.

**Methods:**

Isolated erythrocytes from the healthy volunteers were partitioned into various groups (6 replicates/group) and treated by various concentrations of Al (3–100 *µ*M) in the presence and absence of vit C (0.6 mM) and NAC (1 mM). After 24 hours of treatment, hemolysis was determined from hemoglobin levels in the supernatant. Flowcytometric methods were applied to measure PSE, cell shrinkage, Ca^2+^ content, and ROS abundance using annexin V-binding, forward scatter, Fluo_3_-fluorescence, and DCFDA dependent fluorescence, respectively. Reduced glutathione (GSH) was measured by the ELISA method.

**Results:**

The results showed that a 24 hours' exposure of the erythrocytes to Al (10–100 *µ*M) significantly increased hemolysis in a dose and Ca^2+^dependent manner. Al also dramatically decreased forward scatter. The percentage of PSE cells, Fluo_3_-fluorescence, and DCFDA fluorescence were increased by Al. Furthermore, cotreatment with NAC inhibited the effect of Al on hemolysis, eryptosis, and ROS production. Vit C decreased Al-induced ROS production. However, increased Al-induced eryptosis. There were no significant changes in glutathione after the ALCL_3_ treatment.

**Conclusions:**

Al-induced eryptosis and hemolysis through triggering oxidative stress, while NAC could diverse this effect. In contrast, vit C might intensify Al-induced eryptosis at particular doses through a less known mechanism.

## 1. Introduction

Due to high abundance in the Earth's crust and widespread use in daily life, the exposure of humans with Aluminum (Al) through occupational exposure, foods, drinking water, drugs, and cosmetic products has been increased [1]. Human and animal studies have depicted that Al led to pathological alteration in the structure and function of the various tissues. In the brain, the adverse influences of Al on the synthesis of neurotransmitters and synaptic transmission, posttranslational modification, degradation of proteins, and expression of genes have been demonstrated [2, 3]. Al could also inhibit the mitochondrial electron transport chain and energy production in the liver. Furthermore, Al administration caused a significant decrease in the activity of antioxidant enzymes and the induction of oxidative stress in the hepatocytes [4]. Disturbances in the metabolism of phosphate and calcium in the bone and induction of osteomalacia [5], degeneration of the renal tubular cells [6, 7], and adverse effects on reproductive tissues were other reported toxic effects of Al [8]. Al has also revealed toxicity for the hematological system [9]. Inhibition of hemoglobin biosynthesis [10], alteration in membrane integrity, and stimulation of eryptosis [11, 12] were among possible mechanisms that may have a role in the Al-induced anemia. Eryptosis is defined as suicidal cell death of the erythrocytes and has similar hallmarks to apoptosis in nucleated cells. In this phenomenon, activation of membrane Ca^2+^ channels and increase in cytoplasmic Ca^2+^ led to several modifications including cell shrinkage and translocation of phosphatidylserine (PS) from intracellular to extracellular leaflet of the cell membrane. Despite the cytoplasmic Ca^2+^ activity, eryptosis could be stimulated as a result of increase in ceramide, oxidative stress, energy depletion, and activated caspases as well. Externalization of PS mediated phagocytosis of erythrocyte by the macrophages and their clearance from the blood circulation can lead to anemia [13, 14]. The role of enhanced eryptosis in the pathogenesis of anemia associated disorders including sickle cell disease, thalassemia, and glucose-phosphate dehydrogenase deficiency have been demonstrated as well [15]. Lead [16], mercury [17], and gold [18] are among metal ions that can induce eryptosis. Although several mechanisms may underlie the initiation of eryptosis, an increase in oxidative stress is among the most cited mechanisms. One of the pieces of evidence in this issue is the protective impacts of several synthetic and natural antioxidants on this phenomenon [19]. Vitamin C (vit C) is an antioxidant vitamin that is generally used for ameliorating the adverse effects of toxins [20], cancer [21], and other conditions associated with enhanced-oxidative stress. It has been revealed that vit C can ameliorate the toxic effects of Al [22–24]. To the best of our knowledge, the effect of vit C supplementation against Al-induced hemolysis and eryptosis has not been studied. Therefore, the present study was designed to explore the possibility of Al-induced eryptosis, which might be ameliorated by vit C or N-acetylcysteine (NAC). To this end, the probable protective effect of vit C and NAC on ALCL_3_ induced hemolysis and eryptosis-related characteristics including PS exposure and oxidative stress was determined on the erythrocytes.

## 2. Materials and Methods

### 2.1. Chemicals

Ringer solution (pH 7.4) was prepared from NaCl (125 mM), KCl (5 mM), MgSO_4_ (1 mM), CaCl_2_ (1 mM), glucose (5 mM). In Ca^2+^-free Ringer, CaCl_2_ (1 mM) was substituted by a similar amount of EGTA (1 mM). AlCl_3_ and other chemical reagents were purchased from Sigma, USA.

### 2.2. Assay of Hemolysis

The ex vivo hemolysis assay was conducted on the erythrocytes obtained from healthy volunteers (6 replicates/group) whom informed consent. The study has been approved by the ethics committee of Fasa University of Medical Sciences. In brief, erythrocytes (0.4% hematocrit) were incubated at 37°C for 24 hours in the absence (control group) or presence of AlCl_3_ (3–100 *µ*M). The samples were centrifuged (3 min at 400 ×g, RT) and the supernatants were harvested. Thereafter, the absorbance of hemoglobin (Hb) in the supernatants was determined at 405 nm. 100% hemolysate in hemolysis tests was prepared by mixing the erythrocyte solution with distilled water. As a measure of hemolysis, the percent of hemolysis in each tube is calculated by dividing the absorbance of the test tube by the absorbance of 100% hemolysis [25].

### 2.3. Flowcytomteric Assay for Annexin V-PE Binding and Forward Scatter

Flowcytometry was used to quantify the percent of Annexin V-PE positive erythrocyte and also to measure forward scatter [25]. In brief, after 24 hours of incubation, cells were washed in Ringer solution. Erythrocytes were then stained with Annexin V-PE (EXBIO Praha, Czech Republic) at 1 : 500 dilutions. After 15 min of incubation in the dark, samples were quantified using flowcytometric analysis (FACS Calibur from Becton Dickinson; Heidelberg, Germany). Cells were analyzed by forward scatter and annexin-V-fluorescence intensity was measured in fluorescence channel FL-2 with an excitation wavelength of 488 nm and an emission wavelength of 580 nm.

### 2.4. Measurement of Intracellular Ca^2+^

Intracellular Ca^2+^ was measured using Fluo_3_/AM flowcytometry [25]. In brief, erythrocytes were washed in Ringer solution and then loaded with Fluo_3_/AM (Calbiochem; Bad Soden, Germany) in Ringer solution containing 5 mM CaCl_2_ and 2 *µ*M Fluo_3_/AM. The cells were incubated at 37°C for 20 min and washed twice in Ringer solution containing 5 mM CaCl_2_. After that, the Fluo_3_/AM loaded erythrocytes, which were resuspended in 200 *µ*l, Ringer. Ionomycin (1.0 *µ*M for 30 min) was used as a positive control, though. Moreover, Ca^2+^-dependent fluorescence intensity was determined in fluorescence channel FL-1.

### 2.5. Reactive Oxygen Species (ROS) Assay

2′, 7′-dichlorodihydrofluorescein diacetate (DCFDA, Sigma) was used in order to determine the role of oxidative stress in Al-induced eryptosis fluorescent ROS assay [25]. In this assay, DCFDA was oxidized in the presence of ROS and becomes green fluorescent. In brief, erythrocytes were incubated with AlCl_3_ (100 *µ*M) in the presence and absence of vit C (0.6 mM) or NAC (1 mM). After the treatment, 150 *μ*l suspension of erythrocytes was washed in Ringer solution and stained with DCFDA (10 *μ*M in Ringer solution) at 37°C for 30 min in the dark. After washing with the Ringer solution, the DCFDA-stained erythrocytes were resuspended in 200 *μ*l Ringer solution and DCFDA fluorescence intensity was measured in the FL-1 channel at an excitation wavelength of 488 nm and an emission wavelength of 530 nm on a FACS Calibur (BD). The geomean of the DCFDA dependent fluorescence was then determined as well.

### 2.6. Determination of Reduced Glutathione (GSH)

The level of erythrocytes glutathione (GSSG and GSH) was evaluated using Glutathione Assay Kit (Cayman Chemicals, IBL Hamburg, Hamburg, Germany) according to the manufacturer's protocol. In brief, erythrocytes were washed twice in PBS, incubating for 24 h at 37°C in Ringer solution in the absence or presence of different concentrations of Al^3+^ (3–100 *μ*M). Following that, the erythrocytes were then washed in PBS. Then, 50 *μ*l of the erythrocyte pellet was lysed in 200 *μ*l distilled water, centrifuged at 14,000 × g, and 150 *μ*l of the supernatant was deproteinated by adding 150 *μ*l metaphosphoric acid (10%). GSSG and GSH were then measured with the Glutathione Assay Kit (measured via ELISA reader within the wavelength of 405–414 nm).

### 2.7. Statistical Analyses

SPSS statistical software (SPSS, Chicago, IL, USA, version 21) was applied to analyze the data. Normal distribution of data was checked using the Shapiro–Wilk test (*P* < 0.05). Nonparametric Kruskal–Wallis test was used for the comparison of data between control and experimental groups. The data are represented as mean ± standard deviation (SD) of at least three independent experiments. *P* < 0.05 was considered to be statistically significant.

## 3. Results

### 3.1. The Effect of Al on Erythrocytes Hemolysis

**Figure 1:**
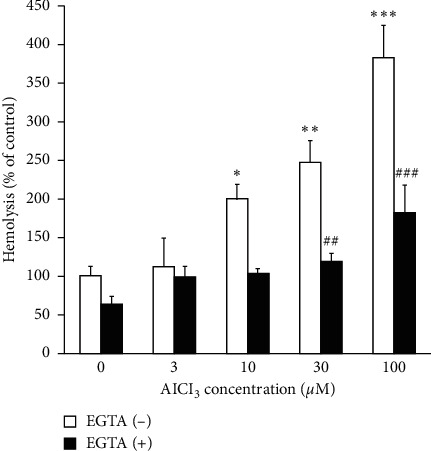
Effect of AlCl_3_ on hemolysis in the absence and presence of EGTA. AlCl_3_ increased hemolysis in a dose-dependent manner. EGTA (1 mM) decreased the effect of Al on erythrocyte hemolysis. Data are mean ± SD of at least five independent experiments. Nonparametric Kruskal–Wallis test was used for the statistical analyses. *P* < 0.05 was considered as significant statistical differences between the group. *P* values compared to the control untreated control group:^*∗*^*P* = 0.028, ^*∗∗*^*P* = 0.002, ^*∗∗∗*^*P* = 0.0001, and *P* values compared to corresponding AlCl_3_-treated groups: ##*P* = 0.027, ###*P* = 0.045.

To explore the effect of Al on hemolysis, erythrocytes were treated with AlCl_3_ (3–100 *µ*M) for 24 hours and the percentage of hemolysis was determined from the absorbance of hemoglobin in the supernatant. As illustrated in Figure 1, the treatment of erythrocytes with various concentrations, from 10 to 100 *µ*M, of AlCl_3_ increased the level of hemolysis in a dose-dependent manner compared to that of the control group, while 3 *µ*M concentration of AlCl_3_ had no significant effects on hemolysis compared to control group.

**Figure 2:**
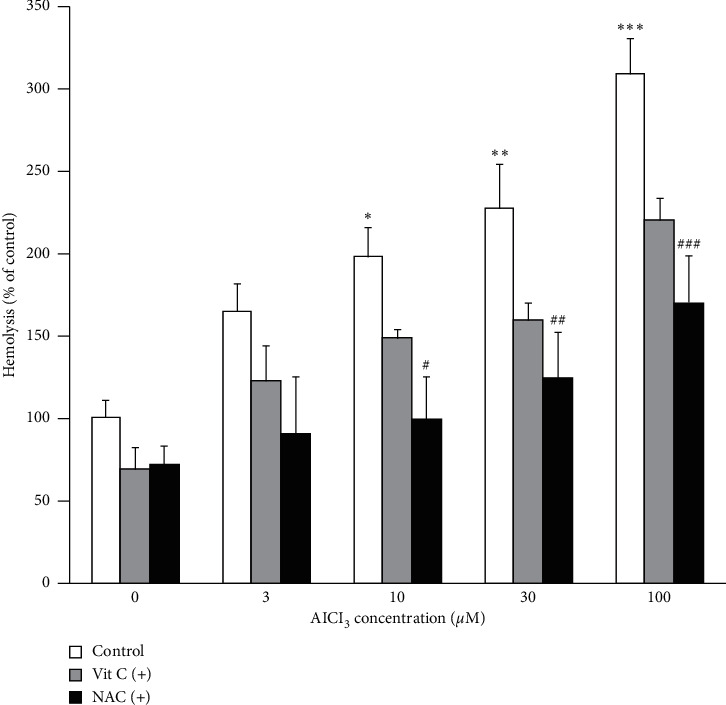
Effect of vitamin C (+vit C) and n-acetyl cysteine (+NAC) on Al-induced hemolysis. AlCl_3_ increase hemolysis in a dose-dependent manner. NAC (1 mM) decreased Al-induced hemolysis while vit C (0.6 mM) had no significant effects on Al-induced hemolysis. Data are mean ± SD of at least five independent experiments. Nonparametric Kruskal–Wallis was used for the statistical analyses. *P* < 0.05 was considered as significant statistical differences between the group. *P* values compared to the untreated control group (^*∗*^*P* = 0.028, ^*∗∗*^*P* = 0.002, ^*∗∗∗*^*P* = 0.0001), significant differences compared to corresponding AlCl_3_-treated groups ((#*P* = 0.031, ##*P* = 0.035, ###*P* = 0.038).

To explore whether the entry of extracellular Ca^2+^ to the erythrocytes was a prerequisite for Al-induced hemolysis or not, erythrocytes were incubated for 24 hours with various concentrations of AlCl_3_ in the presence of 1 mM of EGTA, as a calcium chelating compound. As can be seen in Figure 1, EGTA significantly decreased the effect of AlCl_3_ on hemolysis to that of control cells. Thus, Al may induce hemolysis by the stimulation of extracellular Ca^2+^entry. Further experiments were performed to examine the possible impacts of antioxidants including vit C and NAC, on Al-induced hemolysis. To this end, erythrocytes were treated with AlCl_3_ in the absence and presence of vit C (0.6 mM) or NAC (1 mM). As demonstrated in Figure 2, NAC treatment decreased the level of Al-induced hemolysis significantly (Figure 2), suggesting the possible role of oxidative stress in Al-induced hemolysis. Although, cotreatment with vit C partially decreased the percent of hemolysis in Al-treated cells; no significant effects were observed compared to that of the cells treated with AlCl_3_ alone. vit C at the higher concentrations (>1 mM) significantly increased the level of hemolysis compared to the control group.

### 3.2. The Effect of Vit C and NAC on Al-Induced Eryptosis

**Figure 3:**
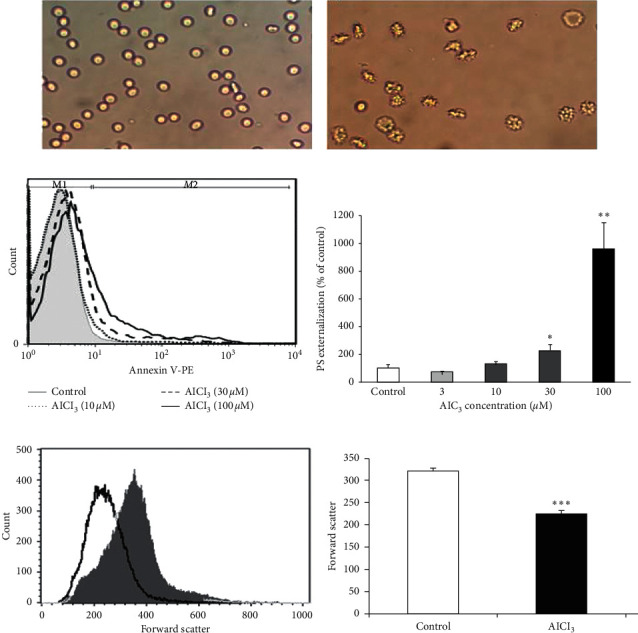
AlCl_3_ induced membrane blebbing, PS externalization, and cell shrinkage in the erythrocytes. Micrographs showed normal shape erythrocytes in the control group (a) and membrane blebbing following treatment of erythrocytes with Ringer solution containing AlCl_3_ (30 *µ*M) (b). Incubation with AlCl_3_ (30 and 100 *μ*M) increased annexin V positive cells (c) and (d). Forward scatter in the erythrocytes ((e) and (f)) following exposure for 24 hours to Ringer solution (control; grey area), AlCl_3_ (100 *μ*M; black line).

**Figure 4:**
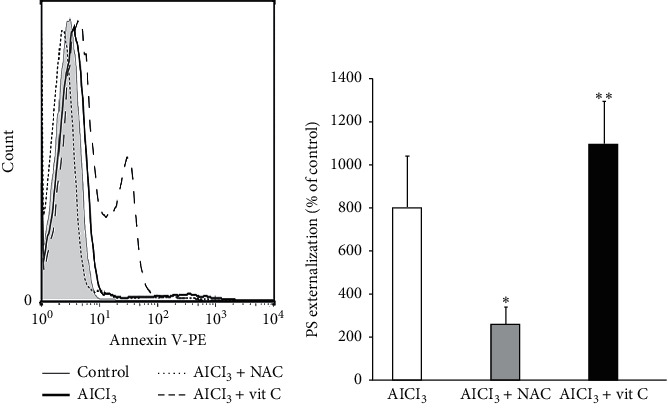
The effect of vit C and NAC on Al-induced PS externalization using Annexin V-PE flowcytometric methods. (a) Histogram of PS externalization of erythrocytes following exposure for 24 hours to Ringer solution (control; grey area), AlCl_3_ (100 *μ*M; black line), AlCl_3_ (100 *μ*M) and vit C (0.6 mM; dashed line), AlCl_3_ (100 *μ*M), and NAC (1.0 mM; dotted line). (b) Arithmetic means ± SD of the percentage of erythrocyte with enhanced PS externalization following treatment with AlCl_3_ in the presence and absences of vit C (0.6 mM) and NAC (1 mM) in Ringer solution for 24 hours. ^*∗*^ and ^*∗∗*^*P* < 0.05 significant difference compared to the AlCl_3_-treated group.

Eryptosis is characterized by three main hallmarks including membrane blebbing, cell shrinkage, and PS externalization. As can be seen in Figure 3(b), a 24 hours' exposure to AlCl_3_ induced membrane blebbing in the erythrocytes. Furthermore, AlCl_3_ (30 and 100 *µ*M) increased the percentage of annexin V positive cells compared to the control group (Figures 3(c) and 3(d)), suggesting PS externalization by AlCl_3_. AlCl_3_ also significantly decreased the forward scatter compared to that of the control group (*P* < 0.001). All these pieces of evidence strongly suggest that AlCl_3_ induced eryptosis in the erythrocytes. NAC cotreatment (1 mM) blunted the effect of Al (100 *µ*M) on PS externalization (Figure 4(b)). Unexpectedly, treatment with vit C alone significantly increased PSE compared to the control group. Furthermore, cotreatment of vit C with Al significantly augmented the effects of Al on the induction of PS externalization.

### 3.3. The Effect of Al on Intracellular Co-Ca^2+^ Concentration Using the Fluo_3_-Fluorescence Method

**Figure 5:**
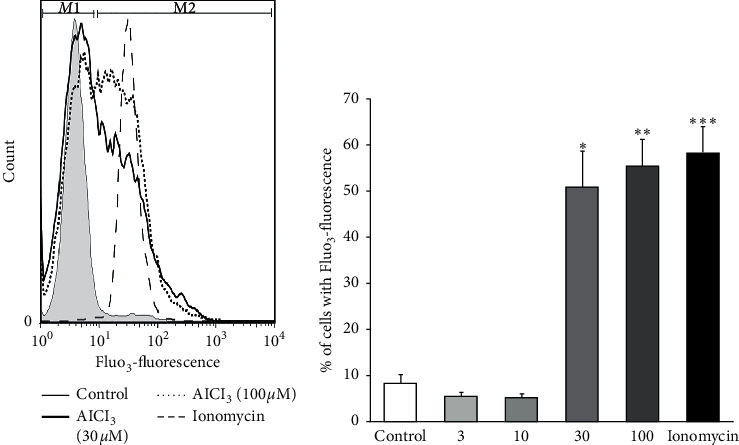
The effects of various concentrations of AlCl_3_ on intracellular Ca^2+^ levels in the erythrocytes. (a) Histogram of Fluo_3_-fluorescence of the erythrocytes following exposure for 24 hours to Ringer solution without (control; grey area) and with the presence of 30 *μ*M (black line) and 100 *μ*M of AlCl_3_*μ*M (dotted line) and 1 *µ*M of ionomycin (positive control; dashed line). (b) Arithmetic means ± SD of the percentage of erythrocyte with enhanced Fluo_3_-fluorescence following treatment with various concentrations of AlCl_3_ and ionomycin (1 *µ*M), in Ringer solution for 24 hours. ^*∗*^, ^*∗∗*^, and ^*∗∗∗*^*P* < 0.001 significant difference compared to control groups.

An increase in intracellular Ca^2+^ concentration could trigger eryptosis. In this study, the Fluo_3_ flow cytometry method was used in order to test whether Al could alter the intracellular concentration of Ca^2+^. As can be seen in Figure 5, ionomycin (1 *µ*M), as a positive control, increased the percent of Fluo_3_-fluorescence cells. Furthermore, incubation of erythrocytes for 24 hours with AlCl_3_ at 30 and 100 *µ*M concentrations increased the percent of Fluo_3_ florescence cells significantly compared to the control group. However, AlCl_3_ at the concentration of 3 and 10 *µ*M had no significant effects on intracellular Ca^2+^.

### 3.4. The Effect of Al on Reactive Oxygen Species Production Using DCFDA Fluorescence Method

**Figure 6:**
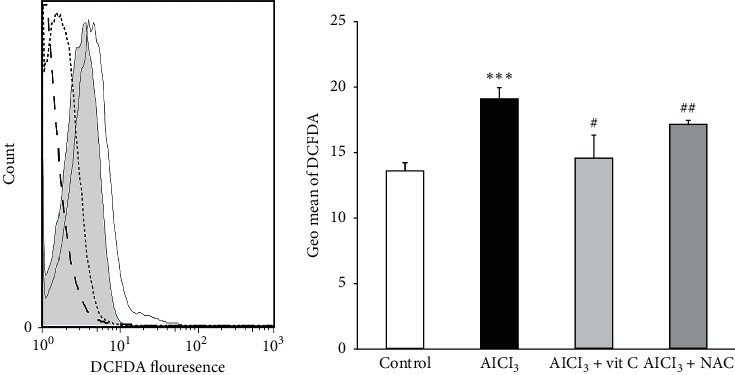
The effects of AlCl_3_ on the formation of reactive oxygen species. (a) Original histogram of DCFDA fluorescence in erythrocytes following exposure for 24 hours to Ringer solution (control, grey areas), AlCl_3_ (100 *µ*M, black lines) AlCl_3_ (100 *µ*M) in the presence of vit C (0.6 mM, dotted lines) and NAC (1 mM, dashed lines). (b) Geo means ± SD of DCFDA fluorescence in erythrocytes after a 24 hours' treatment with AlCl_3_ (100 *µ*M) in the presence of vit C (AlCl_3_ + vit C) and NAC (AlCl_3_ + NAC). ^*∗∗∗*^*P* < 0.001 compared to the control group, ^#^*P* < 0.01 compared to the AlCl_3_ group, and ^##^*P* < 0.05 compared to the AlCl_3_ group.

Reactive oxygen species (ROS) can cause eryptosis. Therefore, the effects of Al on ROS production and possible ameliorating effects of vit C and NAC on this phenomenon were estimated by DCFDA flowcytometry. As illustrated in Figure 6, a 24 hours' exposure to 100 *µ*M concentration of AlCl_3_ significantly increased the DCFDA fluorescence of erythrocytes. Moreover, treatment of erythrocytes with AlCl_3_ (100 *µ*M) in the presence of vit C (0.6 mM) and NAC (1 mM) significantly decreased DCFDA flowcytometry, suggesting a possible role of oxidative stress in the Al-induced eryptosis.

### 3.5. The Effect of Al on Glutathione Level in the Erythrocytes

**Figure 7:**
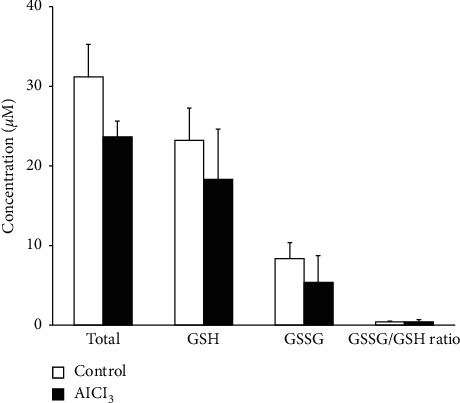
The effects of AlCl_3_ on erythrocyte glutathione level. Total glutathione, reduced glutathione (GSH), and oxidized glutathione (GSSG) were determined in erythrocytes following 24 hours' exposure to AlCl_3_ (100 *µ*M). No significant differences were found between the control and AlCl_3_-treated group.

As depicted in Figure 7, a 24 hours' exposure to 100 *µ*M concentration of AlCl_3_ had no significant effects on total glutathione, GSH, GSSG, and GSSG/GSH ratio compared to the control group.

## 4. Discussion

The morphological and biochemical alteration occurred following the 24 h exposure of erythrocytes with Al including membrane blebbing, cell shrinkage, increase in the PS externalization, increase in intracellular Ca^2+^ level, and elevation of ROS levels, in order to provide clear evidence that Al can trigger eryptosis of erythrocytes in a dose-dependent manner. In addition, we have found that NAC attenuated Al-induced accumulation of ROS and inhibited the Al-induced eryptosis and hemolysis. Although vit C has decreased Al-induced accumulation of ROS, it would surprisingly have failed to reduce Al-induced hemolysis and also increased Al-induced eryptosis of the erythrocytes.

The critical role of oxidative stress in the induction of eryptosis has been demonstrated in numerous studies [15]. However, contradicting results have been reported about the effects of Al on the oxidative stress status of erythrocytes. While Niemoeller et al. [11] has demonstrated that oxidative stress did not involve Al-induced eryptosis, Vota et al. [12] have represented the chronic exposure of erythrocytes with AlCl_3_ which increased the ROS production and decreased the GSH content of the erythrocytes, suggesting the role of oxidative stress in Al-induced effects. In the present investigation, we observed no changes in GSH level and GSH/GSSG following exposure of erythrocyte for 24 hours with Al, while flowcytometric analyses, using DCFDA fluorescence, showed that the ROS content could increase the following Al exposure. Meanwhile, the findings showed that NAC could inhibit the effects of Al on ROS accumulation, eryptosis, and hemolysis, suggesting that oxidative stress may be a downstream mechanism in the Al-induced hemolysis and eryptosis. Plasma membranes of erythrocytes have a high content of polyunsaturated fatty acid [26]; hence, they are highly sensitive to oxidative stress and lipid peroxidation which disrupts the membrane integrity of erythrocytes and results in hemolysis [27]. The inhibition of Al-induced hemolysis by NAC suggested that observed Al-induced hemolysis may be attributed to excessive ROS production and oxidative stress.

It is well-known that vit C keeps cell components including cell membranes against ROS either directly or indirectly via the regeneration of oxidized vitamin E. However, contradicting results have been reported for the effects of vit C on erythrocytes eryptosis and hemolysis. In a recent study, Shan et al. showed that vit C attenuated H_2_O_2_-induced eryptosis in the erythrocytes of human glucose 6-phosphate deficient patients through inhibiting ROS accumulation [28], whereas other studies demonstrated the hemolytic effects of vit C [29]. In agreement with the well-known antioxidant effects of vit C, the data from the current study revealed that vit C decreased ROS production induced by Al. However, vit C induced PS externalization by itself and caused a drastic increment in Al-induced eryptosis. The exact mechanism by which vit C induced PS externalization has not been clear yet. One possible explanation may be autooxidation of vit C in the presence of Al^3+^ which was associated with the production of cytotoxic substances [21]. Moreover, vit C can readily be oxidized to dehydroascorbate (DHA), which is readily transported into erythrocytes by glucose transporter1 (Glut1), abundant in the membrane of erythrocytes. Intracellular DHA is rapidly converted to vit C by GSH dependent reaction. The depletion of GSH eventually induces oxidative stress in the erythrocytes [21]. Further investigations will be needed to understand the interaction between Al^3+^ and vit C.

In addition to ROS production and induction of oxidative stress, it has been demonstrated that disturbance in Ca^2+^ hemostasis (involved in Al-mediated toxicity) and calcium channel blocker was useful for the amelioration of Al's toxic effects [30]. The findings of the current studies clearly demonstrated that Al increased the accumulation of Ca^2+^ in the erythrocytes while calcium ion chelating agent and EGTA (1 mM) have attenuated the Al-induced hemolysis. Thus the observed effects of Al may be secondary to Ca^2+^ entry from the extracellular space to erythrocytes, which is known to trigger eryptosis.

## 5. Conclusion

In conclusion, the investigation revealed that Al-induced eryptosis and hemolysis of erythrocytes, increased in ROS production, and Ca^2+^ entry to erythrocytes which were underlying mechanisms in Al-induced effects. NAC ameliorated Al-induced hemolysis and eryptosis through its antioxidant effects. On the other hand, vit C did not have similar protective effects on eryptosis. At the same time, it has increased eryptosis at particular doses and suggested that ex vivo vit C was not a suitable compound against Al-induced hemolytic and eryptotic effect. On the contrary, NAC would be a better choice to exert a protective effect on cellular eryptosis.

## Data Availability

The data used to support the findings of this study are available from the corresponding author upon request.
